# Zimbabwe Culture before Mapungubwe: New Evidence from Mapela Hill, South-Western Zimbabwe

**DOI:** 10.1371/journal.pone.0111224

**Published:** 2014-10-31

**Authors:** Shadreck Chirikure, Munyaradzi Manyanga, A. Mark Pollard, Foreman Bandama, Godfrey Mahachi, Innocent Pikirayi

**Affiliations:** 1 Department of Archaeology, University of Cape Town, Cape Town, South Africa; 2 Department of History, University of Zimbabwe, Harare, Zimbabwe; 3 Research Laboratory for Archaeology and the History of Art, Oxford University, Oxford, United Kingdom; 4 National Museums and Monuments of Zimbabwe, Harare, Zimbabwe; 5 Department of Archaeology and Anthropology, University of Pretoria, Pretoria, South Africa; ICREA at the Universitat Autònoma de Barcelona, Spain

## Abstract

Across the globe, the emergence of complex societies excites intense academic debate in archaeology and allied disciplines. Not surprisingly, in southern Africa the traditional assumption that the evolution of socio-political complexity began with ideological transformations from K2 to Mapungubwe between CE1200 and 1220 is clouded in controversy. It is believed that the K2−Mapungubwe transitions crystallised class distinction and sacred leadership, thought to be the key elements of the Zimbabwe culture on Mapungubwe Hill long before they emerged anywhere else. From Mapungubwe (CE1220–1290), the Zimbabwe culture was expressed at Great Zimbabwe (CE1300–1450) and eventually Khami (CE1450–1820). However, new fieldwork at Mapela Hill, when coupled with a Bayesian chronology, offers tremendous fresh insights which refute this orthodoxy. Firstly, Mapela possesses enormous prestige stone-walled terraces whose initial construction date from the 11^th^ century CE, almost two hundred years earlier than Mapungubwe. Secondly, the basal levels of the Mapela terraces and hilltop contain élite solid *dhaka* (adobe) floors associated with K2 pottery and glass beads. Thirdly, with a hilltop and flat area occupation since the 11^th^ century CE, Mapela exhibits evidence of class distinction and sacred leadership earlier than K2 and Mapungubwe, the supposed propagators of the Zimbabwe culture. Fourthly, Mapungubwe material culture only appeared later in the Mapela sequence and therefore post-dates the earliest appearance of stone walling and *dhaka* floors at the site. Since stone walls, *dhaka* floors and class distinction are the essence of the Zimbabwe culture, their earlier appearance at Mapela suggests that Mapungubwe can no longer be regarded as the sole cradle of the Zimbabwe culture. This demands not just fresh ways of accounting for the rise of socio-political complexity in southern Africa, but also significant adjustments to existing models.

## Introduction

With three sites on the UNESCO World Heritage list, and two more on the tentative list, the Zimbabwe culture is easily one of the most remarkable cultural developments of the last 2000 years of the sub-Saharan past. For over a century, the origin and evolution of this complex society continues to ignite the imagination of enthusiasts, scholars and the public in different parts of the world (for example [1], [2], [3], [4], [5], [6], [7], [8], [9]). The Zimbabwe culture refers to the practice of constructing dwellings for the élite either inside dry stone-walled enclosures or on top of dry stone-walled terraces, while simultaneously confining commoners to areas outside the walls [10]. Known as *dzimbahwe* in Shona, this configuration of dry stone walls crystallised an ideology of class distinction and sacred leadership [6].

From early on, the Zimbabwe culture was always an appendage of the mainstream Anglo-American archaeological tradition. Wrapped in this international substrate, debates around the origins of the Zimbabwe culture metamorphosed through distinct phases, largely conditioned by the prevailing knowledge production context [11]. Based on biased racial priorities of the time, Bent [1] and Hall [12] believed that the Zimbabwe culture was not local and indigenous, but was Semitic in ancestry. In response to these absurd theories, in 1905 the British Society for the Advancement of Science seconded the well-respected professional archaeologist David Randall-McIver [3] to solve the origins controversy. On the evidence of mediaeval imports recovered at Khami and Great Zimbabwe, Randall-McIver concluded that the Zimbabwe culture was indigenous. Any lingering doubts were dispelled by the meticulous Caton-Thompson [4] who, after labouring at Great Zimbabwe and smaller settlements, found no evidence of foreign influence.

Summers *et al.* combined radiocarbon dating with ceramic, architectural and stratigraphic sequences to outline the evolution of Great Zimbabwe, at the time believed to be the origin and centre of the Zimbabwe culture [5]. Their five phases of occupation established cultural continuities which indicated that Great Zimbabwe became an important centre after the 11^th^ century CE when the initial walls were built. Further research by archaeologists such as Robinson [13], [14], [15] and Garlake [16], [17] among others, led to the development of a credible culture history of farming communities in southern Zambezia. The Leopard's Kopje culture (CE900–1400) was named after the site of Leopard's Kopje approximately two kilometres north-east of the Khami World Heritage Site, and about 24 kilometres west of the modern city of Bulawayo. This culture was widely distributed from west-central Zimbabwe to north-eastern Botswana, and adjacent areas of northern South Africa. It was evident in the sequence of Great Zimbabwe and appeared at many other sites, such as Taba Zika Mambo [14]. The work of Fouché [18] and Gardner [19] at the two Leopard's Kopje sites of K2 (CE1000–1200) and Mapungubwe (CE1220–1290) situated near the Shashe-Limpopo confluence, exposed material culture and burials of huge significance. Owing to a lack of imposing monumental architecture and a 15^th^ century CE date, Mapungubwe was considered an extension of Great Zimbabwe's influence [6]. Chronostratigraphically, K2 belonged to Leopard's Kopje Phase I, while Mapungubwe is part of Leopard's Kopje Phase II [20]. Garlake [17] carried out perfunctory excavations on top of the heavily terraced Leopard's Kopje site of Mapela Hill, about 90 kilometres north-west of Mapungubwe in south-western Zimbabwe. On the basis of solid *dhaka* houses, abundant glass beads and evidence of social distinction, Garlake concluded that it was a capital of an independent Leopard's Kopje state.

From the 1900s until the late 1970s, attempts to understand the evolution of socio-political complexity were, however, Great Zimbabwe-centric in nature. Based on the available chronological and spatial data, it was assumed and widely accepted that Great Zimbabwe was the cradle of the Zimbabwe culture [6]. Although archaeologists at the time recognised the broad similarities between glass beads and ceramics from Leopard's Kopje sites and those of Periods II and III at Great Zimbabwe, little attention was paid to the possibility that the Zimbabwe culture evolved out of the Leopards' Kopje and was therefore more important than was acknowledged at the time. Great Zimbabwe was considered to be the capital of a very extensive empire stretching from the Indian Ocean to the Kalahari. The hundreds of differently-sized dry stone-walled settlements were accorded the status of provincial, district and ward centres in the super state. As a result, an opportunity to unravel the contribution of the Leopard's Kopje in the development of the Zimbabwe culture was missed. This was achieved by Huffman [7] who coupled cognitive structuralist approaches with archaeological data and radiocarbon dates to argue that spatially, Mapungubwe (CE1220–1290) exhibited the Zimbabwe culture pattern earlier than Great Zimbabwe (CE1300–1450). Based on cultural precedence, Mapungubwe became the origin of the Zimbabwe culture. In accounting for the rise of the Zimbabwe culture, Huffman [7] speculated that the shift in power from K2 (Leopard's Kopje Phase I) to Mapungubwe (Leopard's Kopje Phase II/Early Zimbabwe culture) was concomitant with an ideological shift which crystallised sacred leadership and class distinction on Mapungubwe Hill in the early to mid-13^th^ century CE. Because Mapungubwe was thought to be the only Leopard's Kopje site with evidence of class distinction, it was seen as the capital of southern Africa's first state which flourished until CE1290 [7], [21]. Upon its demise, Mapungubwe was followed by Great Zimbabwe (CE1300–1450), which in turn was succeeded by Khami (CE1450–1820). Southern African archaeologists, like their international counterparts, widely accept this framework [22], [8], [23].

**Figure 1:**
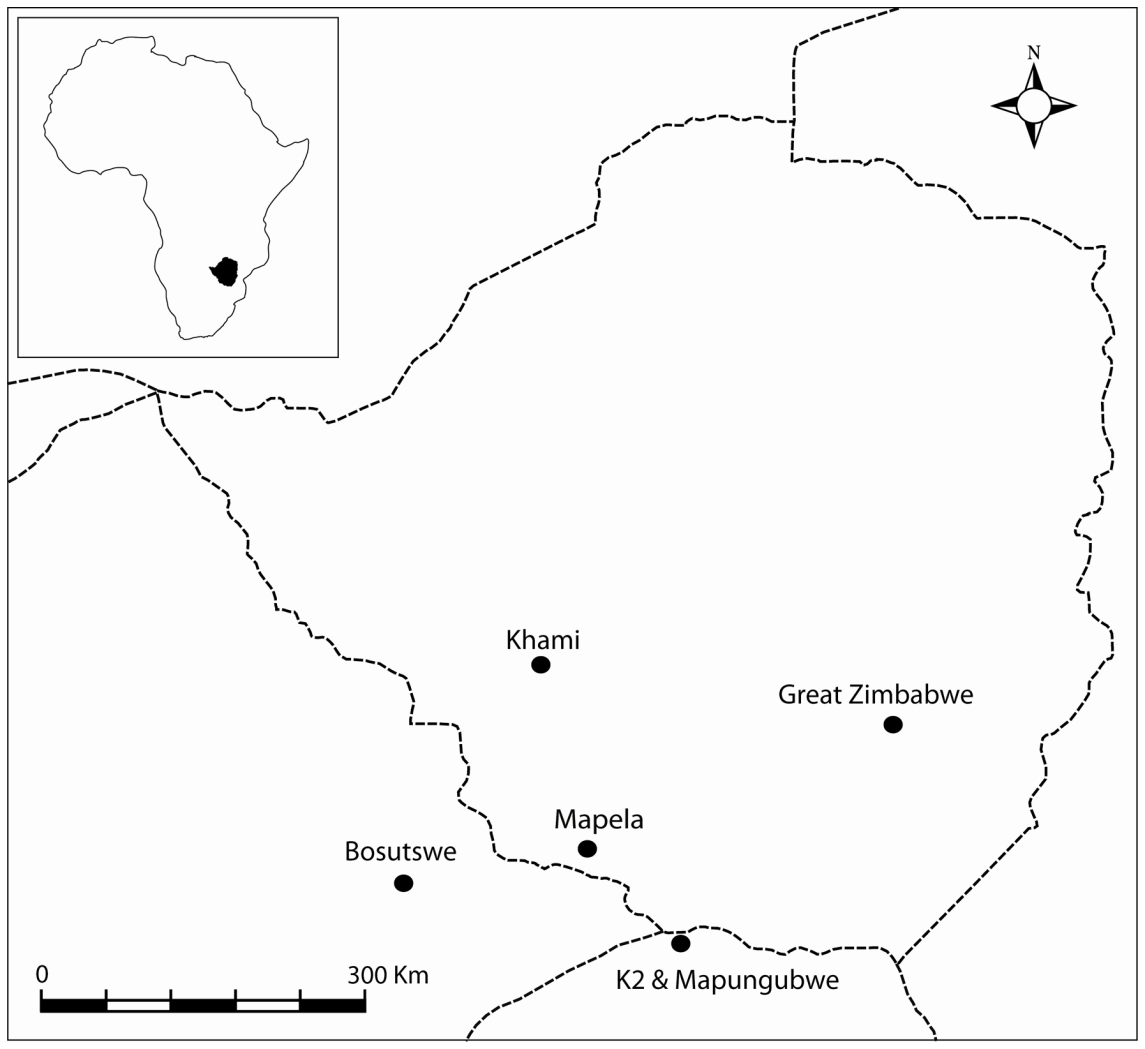
The location of Mapela in relation to other important sites in the region around present-day Zimbabwe.

This entrenched position is, however, problematic because its linearity has caused more important sites to be assumed to be the capitals of the major phases of Leopard's Kopje (Mapungubwe), Great Zimbabwe (Zimbabwe culture) and Khami (Zimbabwe culture). As such, archaeologically well-explored places became the theatres of innovation, thereby editing out of historical significance the many sites that chronologically overlap with the so-called capitals. In fact, sites such as Mapela (Figure 1) were, without much research, granted the status of provincial centres under Mapungubwe (see [24], [8]). As elsewhere in the world, the uneven nature of emphasis and knowledge associated with the linear model became ‘reality’, regardless of how dynamic the past may have been.

To establish the basis for a polycentric model, Chirikure *et al*. [9], [25] reviewed the archaeology of the Leopard's Kopje and its derivative, the Zimbabwe, culture to understand the possible relationships between important and unimportant sites. The main finding was that most sites belonging to the Leopard's Kopje Phase I (Mambo/K2) in south-western Zimbabwe possessed stone walling, *dhaka* floors and participated in long-distance trade [see also Robinson, 15]. Furthermore, there are numerous Leopard's Kopje sites such as Mapela (Garlake, [17]), Mapungubwe [26], Period III levels at Great Zimbabwe [27] and, among others, Malumba [28] which possessed attributes of the Zimbabwe culture in the early second millennium CE. This wider expression of the Zimbabwe culture challenged the assumption that in an approximately one million square kilometre-large southern Zambezia, K2 and Mapungubwe were sole propagators of socio-political complexity.

However, although Chirikure *et al*. [9] suggested that the Zimbabwe culture attributes had already crystallised on the late first and early second millennium CE southern Zambezian landscape, it was based on a re-interpretation of existing data. As such, supporters and sceptics demanded the substantiation of the conclusions with the aid of carefully excavated sites. This provided the motivation for this work – it presents the outcome of detailed research carried out at Mapela in the Shashe region of south-western Zimbabwe.

Mapela was naturally attractive because it contains substantial terrace walls, abundant local pottery, solid *dhaka* floors, and glass and shell beads on the surface – attributes which collectively define the Zimbabwe culture. Geographically, Mapela possesses all the advantages which K2 and Mapungubwe have, and perhaps a lot more. It is situated in rich elephant hunting country close to perennial water sources and is within 20 kilometres of the strategic Gwanda-West-Nicholson gold belt. The fieldwork mapped and excavated the site to establish its spatial extent and the density of stone walling, as well as collecting samples for dating. The results indicated that Mapela is easily the largest known Leopard's Kopje site in southern Africa. The Bayesian chronology, when combined with local pottery and glass bead typologies, suggests that Mapela flourished between CE1055 and CE1400 and therefore pre- and post-dates the generally accepted dates for the flourishing of Mapungubwe (CE1220–1300). Furthermore, it contained prestige stone walls and evidence of class distinction, which suggest that the Zimbabwe culture was expressed earlier at Mapela than at K2 and Mapungubwe. Finally, a polycentric model informed by Actor Network theory, historical data, material culture patterning and modelled Bayesian dates was developed.

### Background to Mapela, Shashe region, south-western Zimbabwe

**Figure 2:**
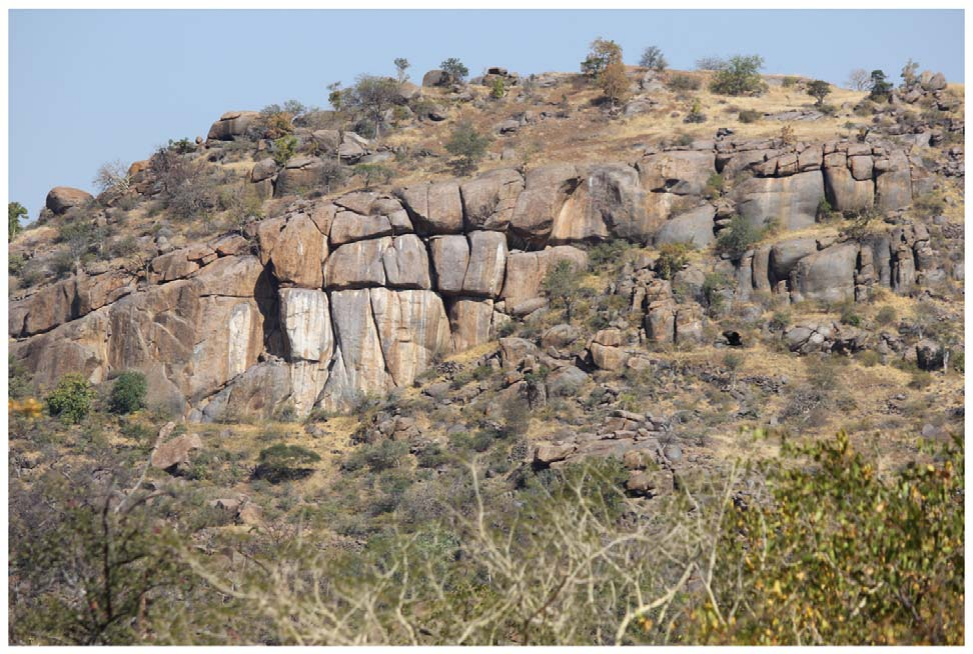
A section of Mapela Hill from the north: All the areas with dry grass (pale yellow in colour) have evidence of human activity.

Mapela (Figure 2) is the name of a prominent hill which lies two kilometres due east of the confluence of the Shashe and Shashani rivers in the south-western Zimbabwe lowveld [17]. As the crow flies, it is approximately two kilometres due north of the Zimbabwe-Botswana border. Mapela is, however, notoriously difficult to access because it lies beyond the major road networks in the area. Because of this inaccessibility, it is not surprising that Garlake [17] is the only known archaeologist to have studied the site before our visit. All interpretations of the site, past and present, are rooted in Garlake's observations. Mapela Hill is characterised by a flat top, with steep cliffs terminating in ledges at different elevations (Figure 2). The resulting flat areas were terraced following the contours of the hill from the bottom to the top, to create spaces for homesteads, some of which were associated with clearly-defined kraals. Amazingly, Mapela is heavily terraced on all sides with revetment walls which often reach up to two and half metres in height.

To establish the cultural affiliation of the site, Garlake [17] test excavated the hilltop area and a midden abutting the north-facing cliff floor. The excavations uncovered typical Leopard's Kopje ceramics, glass beads, fauna, iron work and few copper-based objects. Other finds include spindle whorls and pellets of slag. The hilltop stratigraphic sequence consisted of alternating layers of *dhaka*, or earthen floors, with curved kerbs typical of Great Zimbabwe and related sites. Garlake ([17]: 24) concluded that the glass beads from Mapela were more abundant than any other Leopard's Kopje site in south-western Zimbabwe. Garlake further observed that although stone walling is a characteristic feature of Leopard's Kopje sites, the size and extent of the terraces of Mapela was not approached elsewhere in the region. Therefore, the stone walls of Mapela, according to Garlake, were only dwarfed by those of later sites such as Great Zimbabwe, Khami and others, typical of the flowering of the Zimbabwe culture. Two radiocarbon dates, SR122 and SR115, were obtained from charcoal. However, because of the high error term, the dates are uncertain and when calibrated at 2 sigma, yield a date of between CE1050 and 1400. Nevertheless, they indicate that Mapela was occupied during the Leopard's Kopje period.

**Figure 3:**
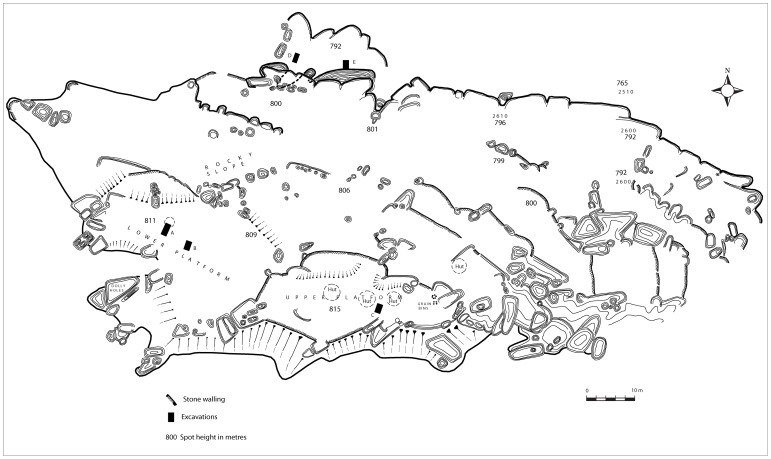
Garlake's map of the summit of Mapela Hill.

After mapping only the summit of Mapela, Garlake ([17]: 2) concluded that the Mapela plateau (Figure 3) is “considerably smaller than Mapungubwe but contains very much more visible stone building”. It is this conclusion which persuaded Africanist archaeologists, despite their never having visited the site, to believe that Mapela was smaller than Mapungubwe. Unfortunately, Garlake did not include the massive terraces on different contours of the hill below the summit. Despite this omission, Garlake concluded that Mapela was a centre of political and economic influence over some considerable area. Furthermore, he noted the presence of class distinction and surmised that further excavation of Mapela, on a more substantial scale, had the potential to fundamentally change perspectives on the development of socio-political complexity in the region.

## Data Collection and Analysis

### Ethical statement

This research was carried out in Zimbabwe. It is an offence to alter, disturb or destroy an archaeological site without written permission from the Executive Director of National Museums and Monuments of Zimbabwe, in terms of the National Museums and Monuments Act No. 25: 11. This research was carried out under Permit no. Mapela 2013/1, issued by the National Museums and Monuments of Zimbabwe. The permit allowed excavation and the export of charcoal samples for radiocarbon dating. No destructive analyses were performed on any other objects which are archived in Harare.

### Mapela fieldwork

Detailed fieldwork was carried out between May and October 2013 in a stepped approach that began with pedestrian surveys, followed by a combination of field and desktop mapping, and ending with stratigraphic excavations.

#### a. Surveying

**Figure 4:**
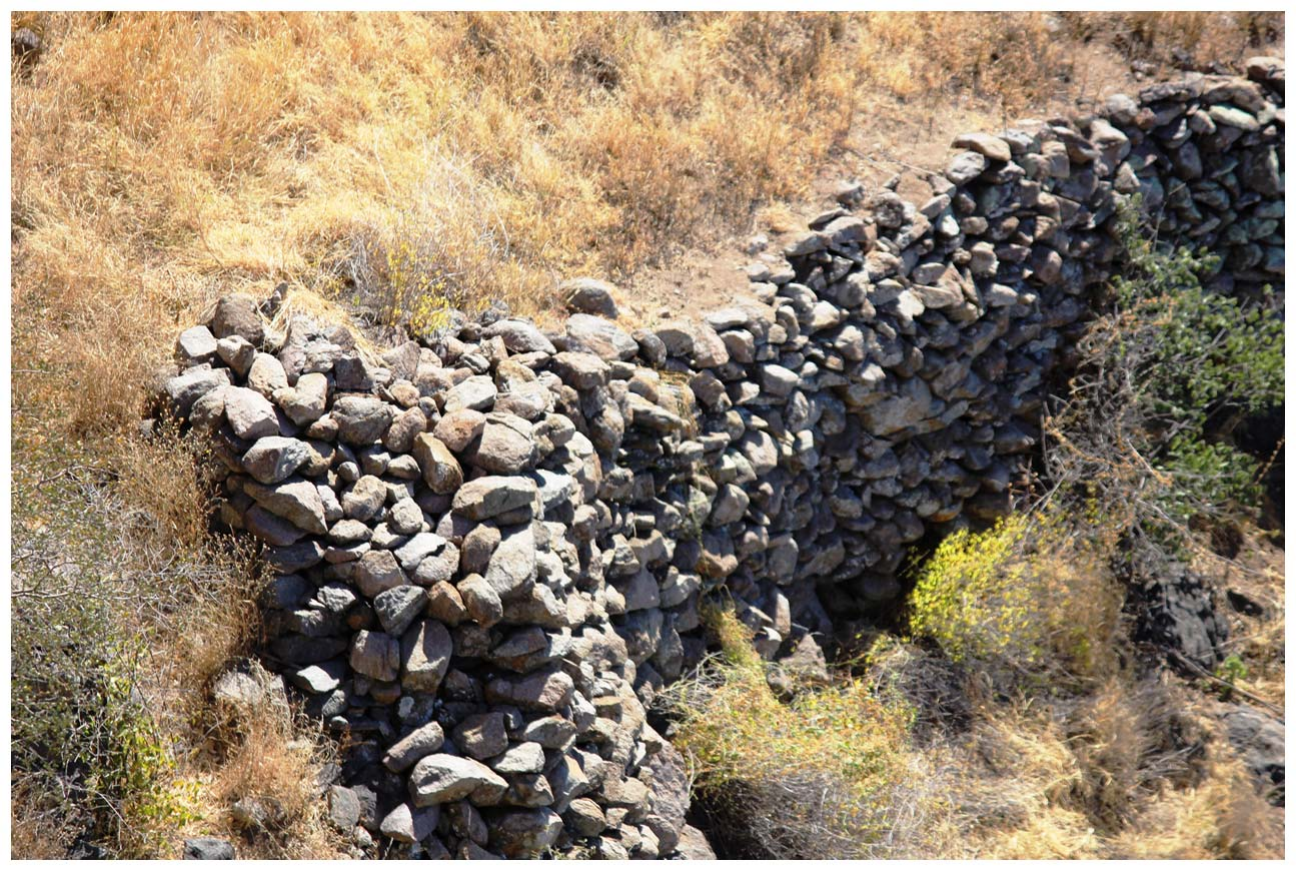
The extensive terrace walls where Excavation Area 2 (Terrace Excavation) (see Figure 5) was situated: Note the buffalo grass on the top section of the terrace and dung clearly visible on the edges without grass.

**Figure 5:**
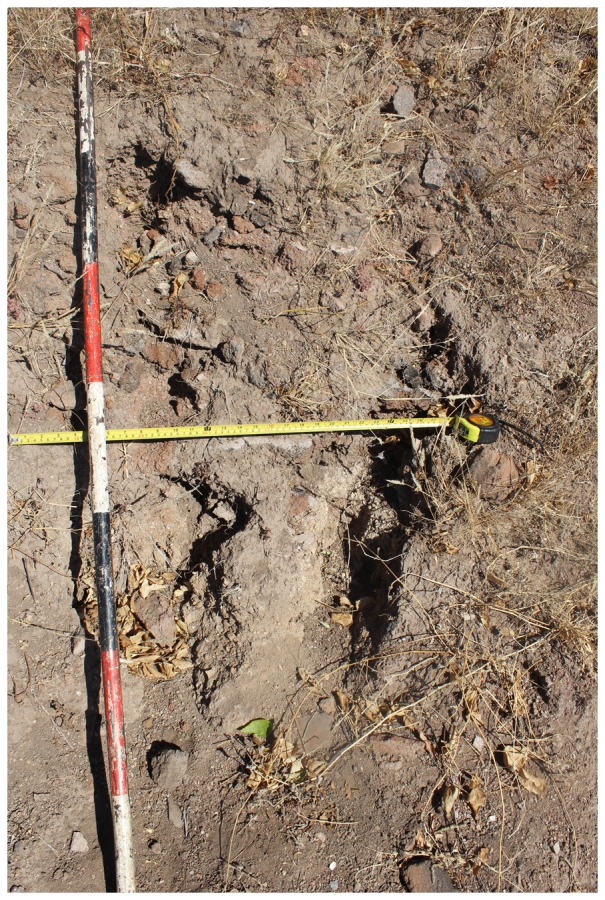
A succession of fired floors on the northern edge of the summit.

**Figure 6:**
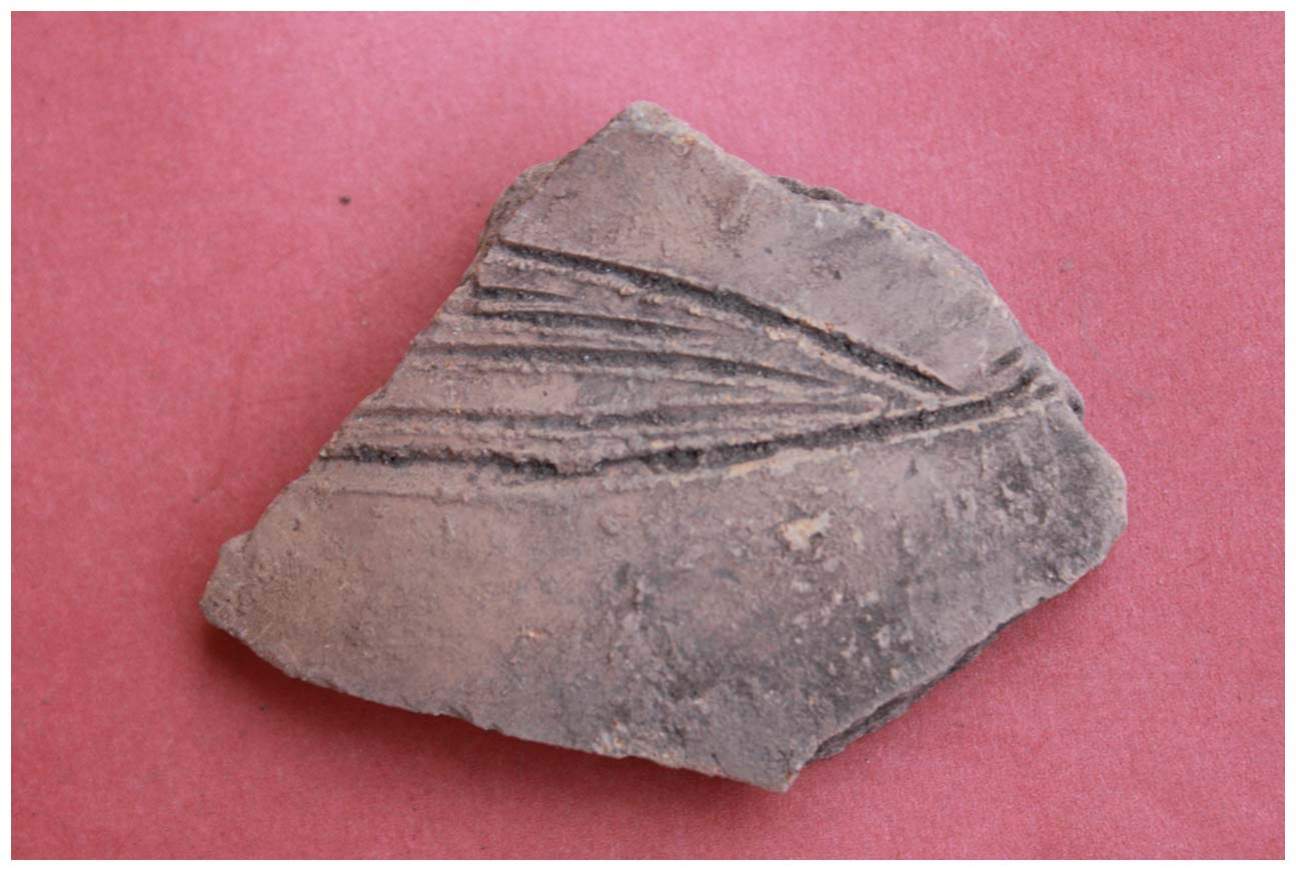
A K2 sherd surface collected from the lower summit of Mapela hilltop.

A dedicated pedestrian survey was conducted across the entire site. In the process, extensive terraces (Figure 4), vitrified dung and middens with a mix of fauna, pottery, metal objects and occasional glass and shell beads were recorded on the flats, terraces and hilltop. On both the terraces and hilltop, erosion often exposed a succession of fired *dhaka* or Zimbabwe cement floors (Figure 5). Surprisingly, for a stone-walled Leopard's Kopje site, vitrified dung was recorded on numerous terraces and flats pointing to the presence of kraals on and below the hill. It has been argued that with the advent of the Zimbabwe culture, cattle kraals were sited away from residential areas (7) but Mapela clearly contradicts such thinking. Surface pottery comprised a mixture of K2 (Figure 6) and Mapungubwe ceramics, with occasional Zhizo sherds. On the western side, the main hill narrows into a smaller kopje which is conjoined to the site of Little Mapela. When these observations are reconciled with Garlake's (17) report, it becomes immediately obvious that only the summit of the main hill was mapped, thereby excluding approximately three quarters of the site. Unfortunately, this underestimation of Mapela's size has given it a subordinate role to Mapungubwe, when the opposite is true if settlement size is the only variable that determines political importance.

**Figure 7:**
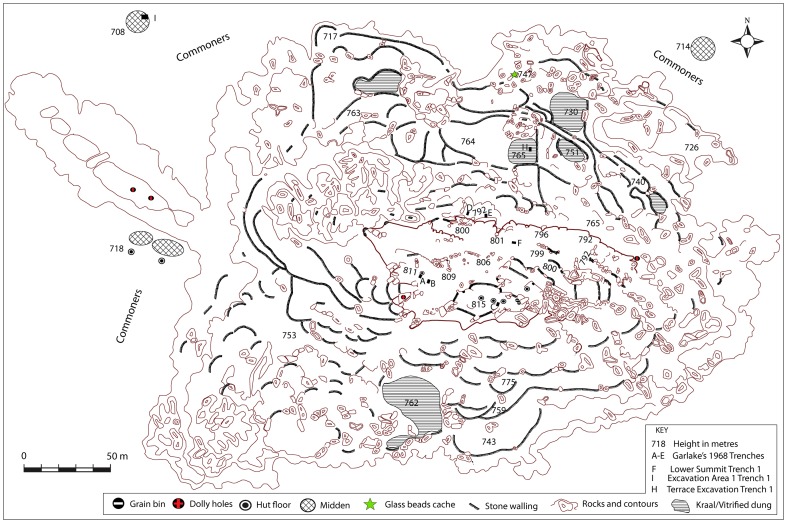
Complete map of Mapela Hill showing the summit, terraces and surrounding flats: Note the density of stone terraces, which by far outnumber those on Mapungubwe Hill.

These far-reaching observations demonstrated that it was impossible to understand the complexity of the site without mapping it in full. Owing to advances in satellite imaging and GIS software, it was possible to use a combination of desk and field based mapping techniques. Sadr and Rodier [29] demonstrated the utility of this approach when they mapped stone-walled sites around Gauteng in South Africa. To begin with, Garlake's map of the summit was superimposed on a Google Earth image of Mapela Hill to establish control points. Once the best-fitting overlay was established, the terraces and other prominent features were screen digitised using GIS software. In cases where opacity was poor, field walking and geocoded GPS recording enabled the capturing of accurate details. Such an endeavour, for the first time, finally produced a complete map of Mapela Hill (Figure 7).

As is clear from the difference between the ground and the summit elevation, Mapela Hill is just over 90 metres high. Apparently, most contours of the hill were heavily terraced indicating that the site was intensively occupied. Furthermore, if the map of the summit is considered in relation to the whole site, it becomes decisively clear that Garlake's descriptions – and all subsequent interpretations based on it – severely underestimated the size of the site.

#### b. Excavation

In order to understand the chronology and material culture of Mapela, stratigraphic excavations were conducted at three areas: (1) Excavation Area 1 on the flats (marked I on Figure 7); (2) Terrace Excavation Trench 1 on a substantial north-facing terrace (marked H on Figure 7); and (3) Lower Summit Excavation Trench 1 on the lower summit (marked G on Figure 7). The intention was to develop an impression of the chronology and activities taking place in different areas of the site – the flats, the terraces and the summit. Such a strategy allowed us to explore the issue of class distinction at the site. Terrace Excavation Trench 1 was located on the eastern edge of a sloping terrace covered by vitrified dung (Figure 4). The lower summit trenches were sited on a midden purposely selected to provide a comparison with Garlake's stratigraphy on the upper platform.

#### c. Stratigraphy

**Figure 8:**
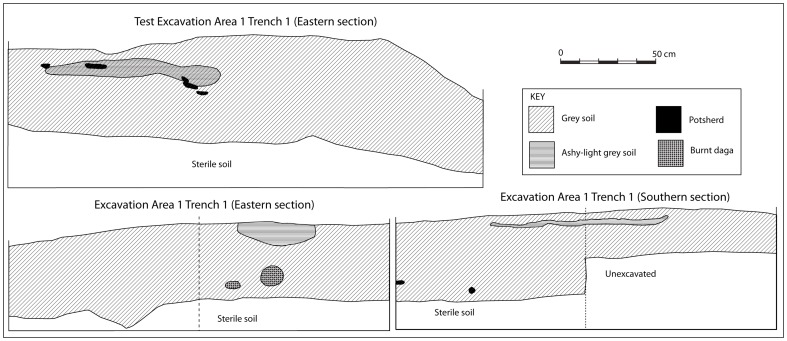
A section of Trench 1, Excavation Area 1 on the northern flats.

The excavations proceeded in 10 centimetre spits. As Figure 8 shows, the stratigraphy of Excavation Area 1 was not more than 50 cm deep and was not clearly defined except in a few cases with a lens of ash. The finds included slag, K2 and Zhizo ceramics, together with a small number of shell beads.

**Figure 9:**
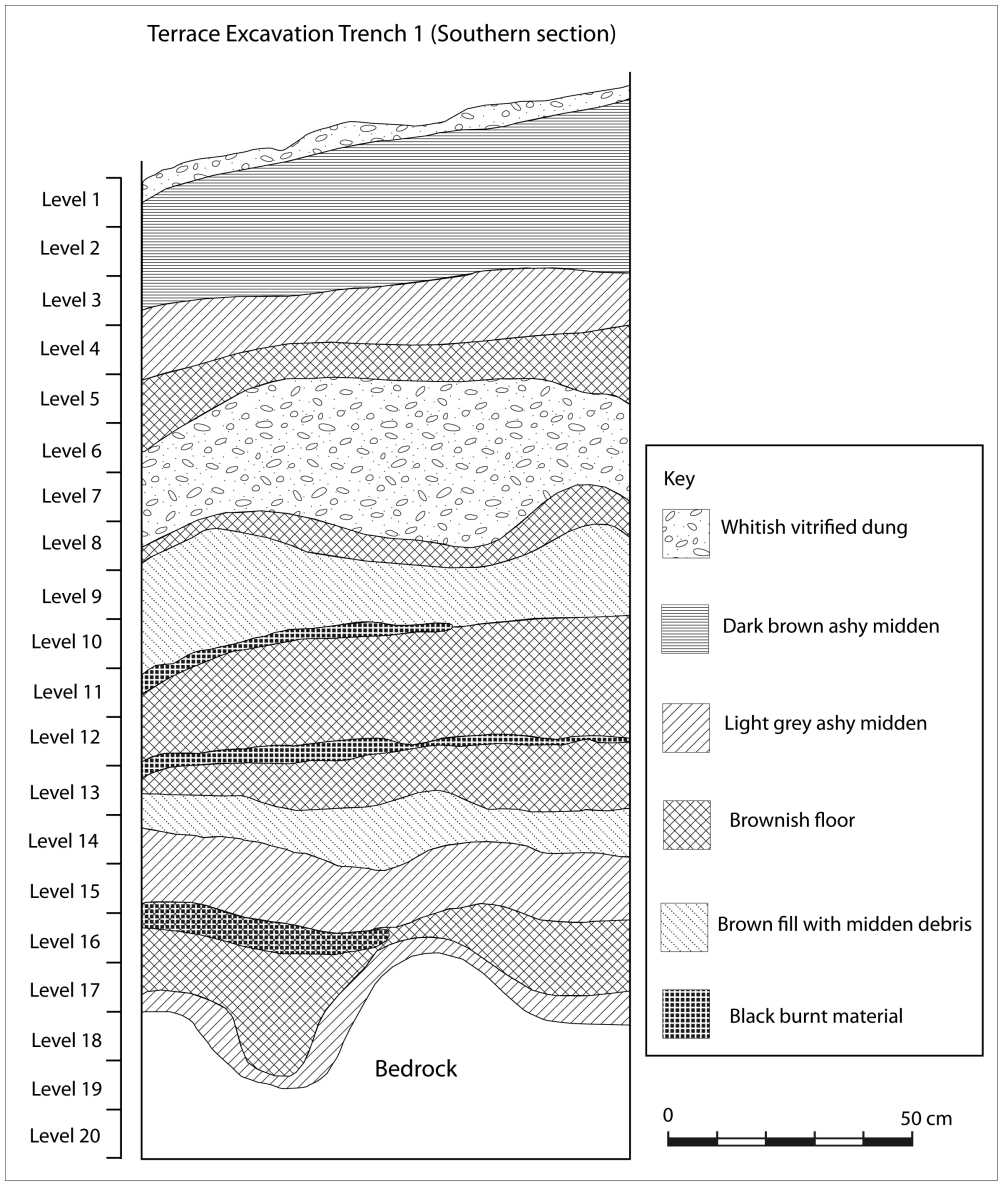
The stratigraphy of Trench 1, Excavation Area 2 on the terrace.

Terrace Excavation Trench 1 yielded an authoritatively informative stratigraphy (Figure 9). A very thin layer of vitrified dung constituted the topmost layer and was followed by two successive middens, one brownish and another greyish in colour. Underneath this was a layer of vitrified dung, followed by another *dhaka* floor which rested on top of a brown fill with midden debris. The fired nature of the floor ruled out other alternatives, such as caps which may result from repeated cattle or animal hoof stamping (see Huffman, [24]). Below the fill was a thin layer of burnt grass, carbonised sorghum seeds, charcoal and *dhaka* in level 11. A very thick *dhaka* floor followed underneath (level 12). It was succeeded by yet another event associated with burning. A mixture of wood charcoal, grass and sorghum seeds was recovered in this level 13. The recovery of sorghum seeds suggests that the circular stone feature that continued from the layer above, on the northern side of the trench, was a grain bin foundation. Below this was another floor underlaid by a layer comprising of broken *dhaka* fragments, pottery and charcoal. This was followed by a midden which accumulated on top of a floor, and covered one side of the trench. The southern corner of the trench contained charcoal, ash and burnt *dhaka* in level 16. It seems that this material collected on top of a floor (levels 17 and 18) which sealed a very thin midden that contained K2 pottery, charcoal and three glass beads (levels 18 and 19). Because of the depth and integrity of this stratigraphy, nine samples of carbonaceous materials from levels 7, 9, and 13 to 19 were submitted for radiocarbon dating.

**Figure 10:**
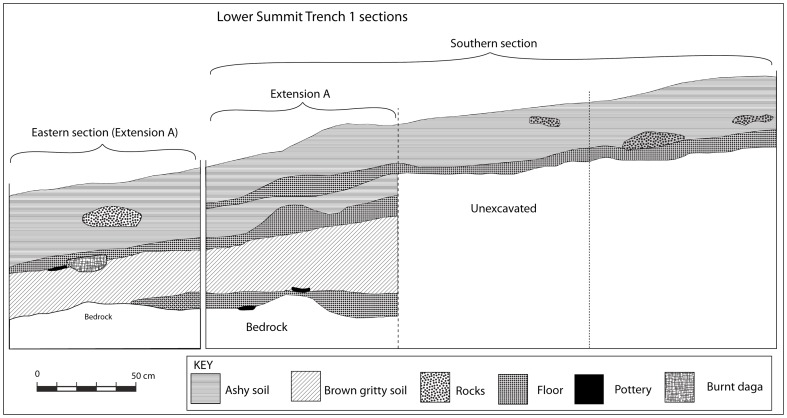
The stratigraphy of Trench 1 on the lower summit.

Lower Summit Trench 1 was just over half a metre deep. However, the stratigraphy was complex, comprising alternating layers of midden and floors. Initially, a 2×1-metre trench was excavated but this was abandoned after 20 centimetres when an intact floor was encountered. A decision was made to extend the trench by one metre (Extension A) and to excavate that to bedrock (Figure 10). Only four more layers were encountered before reaching bedrock. A floor lay directly on top of this bedrock, where a decorated K2 sherd was found.

When combined, the excavations produced a number of significant finds ranging from domestic pottery, spindle whorls, ferrous metal objects, copper-based ornaments, glass beads, fauna, fish bone, carbonised seeds to fragments of *dhaka*.

### 2.2 Analysis and Results

#### 2.2.3 Artefact studies: pottery and glass beads

**Figure 11:**
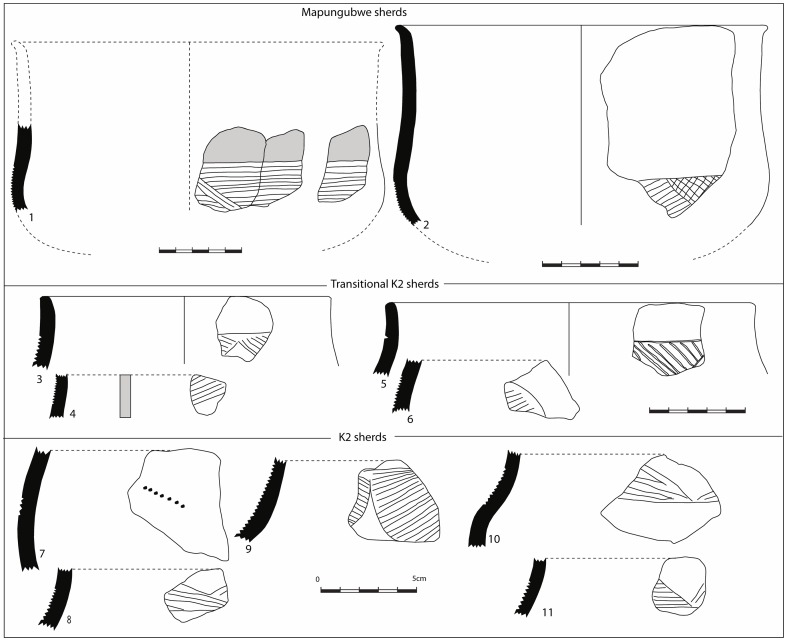
Selected K2, Transitional K2 and Mapungubwe ceramics from Mapela Hill.

Only domestic pottery and glass beads were typologically and stratigraphically analysed to determine the sequence of occupation and the identity of the inhabitants of Mapela. Since the ceramic and bead sequences in southern Africa are reasonably well dated and are reproducible, it is easy to cross-date new sites by comparing their ceramics and beads to established types [21]; [30]. With high levels of stratigraphic integrity, Terrace Excavation Trench 1 provided useful insights for building ceramic and bead sequences on the terrace. The ceramics were analysed using the standard typological technique of considering vessel shape, decoration position, technique and motif (see [21]). The main indication from the typological analysis was that typical K2 pottery (Early Leopard's Kopje) dominates the bottom of the sequence with transitional K2 ceramics in the intermediate layers. Mapungubwe (Late Leopard's Kopje/Early Zimbabwe culture) ceramics became more frequent from the middle to upper layers of the sequence (see Figure 11 for selected illustrations). The same pattern was also mirrored in Lower Summit Excavation Trench 1 where the bottom layers were dominated by K2 pottery, followed by transitional and Mapungubwe pottery. Occasional Zhizo ceramics were recovered in all excavation areas but more work is required to interpret the implications of this association. Therefore, the ceramic sequence at Mapela consists of all the major pottery phases associated with socio-political complexity in the Middle Limpopo Valley – Zhizo, K2, Transitional K2 and Mapungubwe.

The glass beads from Mapela were placed within a chronological and typological framework established by Robertshaw *et al*. [31] and Wood [30]. This classification scheme is based on a meticulous combination of visual and metric attributes of the glass beads, but was also independently verified using geochemical techniques (Table 1). Geochemically, K2 and Mapungubwe beads are distinct which further confirms the macroscopic evidence.

**Table 1:** Characteristics of glass: Early and Late Leopard's Kopje glass beads from southern Africa (after Robertshaw *et al*., 2010).

Bead type	Metric attributes	Colour	Chemistry	Sites where typical beads were recovered
**K2 (CE980**–**1200)**	2–3.5 mm diameter; 1.2–4 mm long	Transparent to translucent blue-green to light green beads	Made of soda-alumina glass of south Asian origin	K2 – South Africa, Zimbabwe Hill, Zimbabwe, Kgaswe – Botswana, Pont Drift – South Africa, Schroda – South Africa, Mapela – Zimbabwe
**Indo-Pacific (CE1000**–**1250)**	2.5–4.5 mm in diameter and cylindrical in shape	Black and brownish-red beads are opaque; yellow, soft orange, green and blue-green ones are translucent	Made of soda-alumina glass of south Asian origin	K2 – South Africa, Zimbabwe Hill – Zimbabwe, Kgaswe – Botswana, Pont Drift – South Africa, Schroda – South Africa, Mapela – Zimbabwe
**Mapungubwe (CE12540**–**1300)**	2–3.5 mm diameter	Opaque black, translucent-opaque glass including blue-green, green, yellow orange, transparent cobalt blue, plum	Made from high-alumina, low- calcium plant-ash glass of south or south-east Asian origin	Taba Zika Mambo – Zimbabwe, Mapela – Zimbabwe, Zimbabwe Hill – Zimbabwe, Mapungubwe – South Africa, Bosutswe – Botswana, Skutwater – South Africa, Khami − Zimbabwe

K2 and Indo-Pacific beads belong to the K2 series; there are no transitional K2 series.

**Figure 12:**
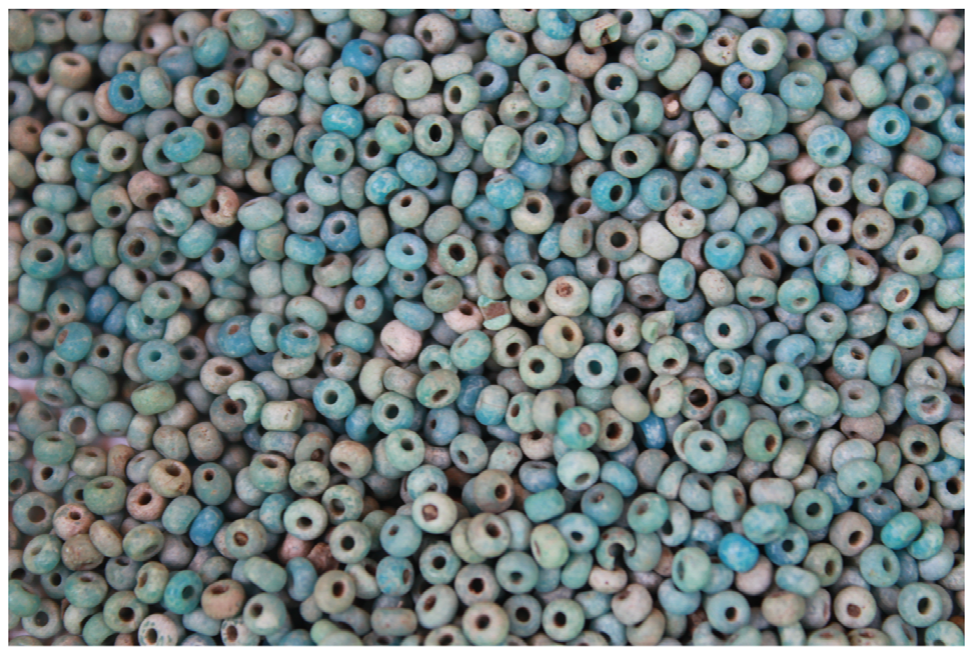
Mapungubwe-type glass beads from the glass bead cache (see Figure 7) on the edge of a lower terrace, northern side of Mapela.

The earliest bead series from the terrace excavation belonged to the K2 series. These were followed by Mapungubwe series glass beads. Apart from being chronological markers, glass beads are also seen as status markers [32]. Over a thousand beads were recovered from the terrace, while far fewer came from Excavation Area 1 and the lower summit. However, a significant amount of glass beads was eroding from several areas. On the northern side, thousands of Mapungubwe glass beads were eroding out of a context which also contained Mapungubwe beakers (labelled glass bead cache on Figure 7). A decision was made to salvage these beads through scraping the surface and sieving the soil (Figure 12). Overall, the glut of glass beads indicates that Mapela was a major player in trading and exchange relationships with the Indian Ocean.

The pottery and glass bead typology indicated that Mapela contains both Leopard's Kopje Phases I and II, or early Zimbabwe culture traits on the terraces, flats and hilltop. However, samples of carbonised seeds and charcoal were radiocarbon dated using AMS and conventional radiocarbon dating techniques to develop an absolute and independent chronology.

#### Chronometric dating: the Bayesian modelled dates

Samples for radiocarbon dating were obtained from charcoal and short-lived samples, such as carbonised seeds and twigs. Table 2 presents the context, laboratory numbers and the dated material.

**Table 2:** Presents the materials dated, their context, and uncalibrated radiocarbon dates.

Laboratory number	Level	Material dated	Uncalibrated dates
Beta-362445 (AMS)	7	Carbonised twigs	770+/−30 BP
Beta-362446 (conventional)	9	charcoal	770+/−30 BP
Beta-362447 (AMS)	13	Carbonised seeds	770+/−30 BP
Beta-362448 (AMS)	14	charcoal	740+/−30 BP
Beta-362449 (AMS)	15	charcoal	820+/−30 BP
Beta-362450 (AMS)	16	charcoal	890+/−30 BP
Beta-362451 (AMS)	17	charcoal	860+/−30 BP
Beta-362452 (AMS)	18	charcoal	830+/−30 BP
Beta-362453 (AMS)	19	charcoal	900+/−30 BP

**Figure 13:**
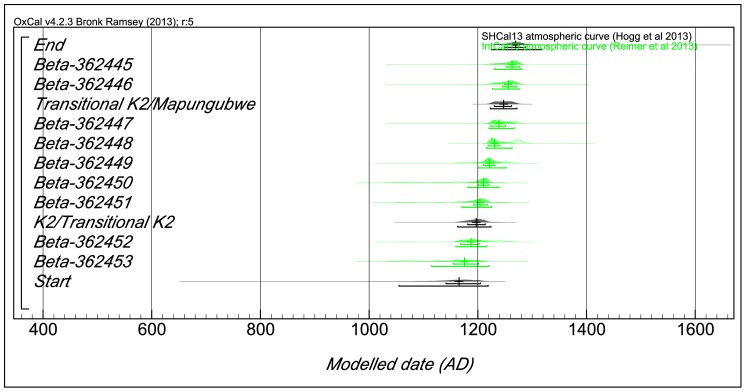
Modelled dates from Levels 7, 9, and 13 to 19, Excavation Area 2 (Terrace Excavation), Trench 1.

The dates were modelled following Bayesian techniques in the software OxCal version 4.2.3 at Oxford University's Research Laboratory for the History of Archaeology and Art. Bayesian models are conditional probabilities which allow for pre-existing information to be incorporated into the current data, to permit the development of an integrated interpretation process [33]. The prior distribution of the unknown parameter Ø is updated, on observing the realised value of the data X, to the posterior distribution, through Bayes' law. Inference about Ø is then extracted from this posterior. The prior is a formal statement of what is known before the process of data collection, while the posterior is the desired outcome. Bayes' theorem relates posterior likelihood X to the prior. Based on the stratigraphy, and the observation that K2 ceramics and glass beads were at the bottom, followed by transitional pottery and Mapungubwe material culture, a sequence model was run in OxCal version 4.2.3 [34], assuming that the dates at the bottom are older than those above. The recommended Southern Hemisphere Calibration Curve (SHCA13) was used as it was developed using dendrochronologically dated wood from the corresponding hemisphere [35], [36]. Because the dates were from a single stratified sequence, all the dates were combined into a single model. The inbuilt SPAN factor in OxCal version 4.2.3 was used to develop the intervals of occupation between K2 and Transitional K2 on the one hand, and Transitional K2 and Mapungubwe on the other. The results are shown in Table 3 and Figure 13.

**Table 3:** Presents the unmodelled and modelled dates for the samples from Mapela.

Name	Unmodelled (BCE/CE)				Modelled (BCE/CE)			
	From	to	%	median	from	to	%	median
End					1225	1317	95.4	1269
Beta-362445	1217	1282	95.4	1254	1230	1281	95.4	1263
Beta-362446	1217	1282	95.4	1254	1227	1276	95.4	1256
Transitional K2/Mapungubwe					1223	1272	95.4	1247
Beta-362447	1217	1282	95.4	1254	1220	1267	95.4	1239
Beta-362448	1224	1291	95.4	1270	1216	1263	95.4	1231
Beta-362449	1165	1265	95.4	1224	1200	1252	95.4	1221
Beta-362450	1041	1218	95.4	1140	1181	1239	95.4	1210
Beta-362451	1049	1256	95.4	1186	1170	1225	95.4	1204
K2/Transitional K2					1163	1224	95.4	1197
Beta-362452	1161	1264	95.4	1214	1159	1216	95.4	1187
Beta-362453	1039	1210	95.4	1123	1115	1220	95.4	1175
Start					1055	1219	95.4	1165
ShCal13								

At the 95% confidence interval, the start of K2 phase was estimated to be from 1055 to 1219 cal CE while the K2/Transitional K2 phase was estimated at between 1163 and 1224 cal CE. The Transitional K2/Mapungubwe boundary has been estimated to between 1223 and 1272 cal CE. The end of the sequence, based on the dates available up to level 7, is between 1225 and 1317 cal CE (95%). The presence of Mapungubwe pottery and glass beads in levels 1 to 6 suggests that the sequence extends to the 14^th^ century. The lack of dates from the top section of the trench was considered unimportant in view of the need to provide a tight sequence relating to the K2, Transitional K2 and Mapungubwe intervals. The dates from Garlake [17] were modelled separately as they are not from the same stratigraphic sequence. The idea was to establish if there is general concordance between the old and new dates. The obvious limitation of Garlake's dates is that they have high uncertainties (caused by huge error terms). The date SR122 calibrates to between 1028 and 1390 cal CE, while SR115 is estimated to be between 1169 and 1435 cal CE. Not much can be made from these dates, but generally they overlap with important parts of our sequence.

The modelled dates authoritatively show that by the late 11^th^ century cal CE, K2 people were established on the and that in the 12^th^ century, Transitional K2 pottery was being produced, with Mapungubwe material appearing towards the middle of the 13^th^ century cal CE.

## Discussion

“*Class distinction and sacred leadership characterised the Zimbabwe culture, the most complex society in precolonial southern Africa. This complex society evolved between AD 1000 and 1300 at the sites of K2 and Mapungubwe in the Shashe-Limpopo Valley*” ([24]:14)

**Figure 14:**
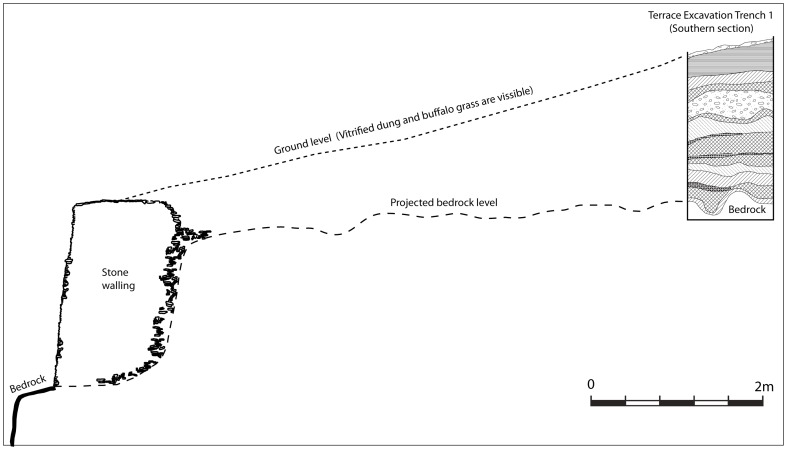
The relationship between the terrace and the stratigraphy of Trench 1 Excavation Area 2.

In discussions of socio-political complexity in southern Africa, the view that Mapungubwe represents the first expression of Zimbabwe culture is so entrenched that it has become accepted lore. And yet recent archaeological work at Mapela has generated insights fundamental for re-envisioning our understanding of the evolution of socio-political complexity in southern Africa. The archaeological work at Mapela decisively reveals that Garlake's description of the site greatly underestimated its size and importance. Crucially, Mapela has K2, Transitional K2 and Mapungubwe ceramics and glass beads in stratified and uninterrupted contexts. The Bayesian chronology dates the earliest occupation of the excavated terrace to the 11^th^ century CE, right at the onset of the K2 period (see [26]). By the mid-11^th^ century CE, K2 people built houses with solid *dhaka* floors on massive stone-walled terraces, a tradition which continued into the Mapungubwe period. An eroded section on the northern edge of the summit shows a sequence of floors from bedrock up to the top of the sequence. As we have seen, the basal layers contain K2 followed by Transitional K2 material. This conclusion is further strengthened by the evidence from Terrace Excavation Trench 1, where a sterile earthy fill underlies the earliest K2 occupation of the terrace on the eastern side of the trench. Triangulation by theodolite indicated that this level also represented the top of the terrace platform on the edge, as illustrated by Figure 14.

The construction of dry stone walls during K2 times is hardly surprising given that Robinson [15] has demonstrated that the Leopard's Kopje communities occupying most of south-western Zimbabwe were building prestige stone-walled terraces from around CE900 onwards (see also [9]). Dry stone walls are the most important defining feature of the élite Zimbabwe culture (the name ‘Zimbabwe’ comes from *dzimbahwe*, meaning houses of stone) pattern [6]; [22]; [8]. The observation that terrace wall construction at Mapela is earlier than the supposed ideological transformations at K2, which are traditionally assumed to have crystallised class distinction at Mapungubwe in the early 13^th^ century, endorses Robinson's conclusion [14], [15]. During this early period, Mapela had a hilltop occupation, a terrace occupation and a flat area occupation consistent with social hierarchy and class distinction. Working on both cultural and chronological logic, the presence of Zimbabwe pattern dry stone walls, at Mapela shows that it was probably far more influential than the chronologically overlapping K2 (which did not have elements of the Zimbabwe culture). On the basis of precedence, Mapungubwe may have copied Mapela which, as we have seen, had a fully-evolved Zimbabwe culture before the Middle Limpopo sites. This evidence from Mapela also supports the observation made by Chirikure *et al.* [9] that dry stone wall construction on raised ground was an established cultural practice during the Leopard's Kopje Phase I (K2/Mambo). More importantly, these terrace walls were also, from the onset, associated with solid *dhaka* floors with curved kerbs, dispelling the notion that they appeared first on Mapungubwe Hill before anywhere else in the region. Huffman [21] argues that the status of the Leopards' Kopje walls and sites in south-western Zimbabwe and adjacent areas of north-eastern Botswana is currently unclear. Given that the Leopard's Kopje people at K2 and Mapungubwe belonged to the same cultural group as the Leopard's Kopje people at Mapela and other places who shared a similar ideology, it can be authoritatively argued that the dry stone walls in south-western Zimbabwe and associated *dhaka* structures represent the earliest Zimbabwe culture expression. Evidence from south-western Zimbabwe shows that everything that makes up the Zimbabwe culture at Mapungubwe appeared earlier at Mapela, making Mapela one of the most important sites with secure evidence of the evolution of the Zimbabwe culture.

Historically and ethnographically, one of the features of élite Zimbabwe culture is the ideology of class distinction crystallised on the ground through building élite dwellings on raised ground and commoner dwellings on the flats [37], [22]; [24]. A variation of this theme involves occupation by the élite of walled areas, while commoners resided outside the walls [6]. The principle of building élite dwellings on terraces is demonstrated at Mapela during K2 times. In fact, the presence of abundant terraces constructed at different elevations further indicates the principle of ranking and class distinction at Mapela, with those on the lower terraces being of a lower class than those above them. The Lower Summit Excavation Trench 1 on the hilltop produced K2 pottery as well as Transitional K2 material in the basal levels, demonstrating an élite occupation of the hilltop from the outset. Fundamentally, Mapela also boasts of an occupation of the surrounding flats by people making initially K2, and later Mapungubwe ceramics, which confirms that class and social differentiation had been established by the 11^th^ century CE. Mapungubwe therefore exhibits cultural practices that were already on the landscape, showing continuity in tradition through interaction, copying, and other means.

Mapela furthermore possesses thousands of glass beads suggesting that it was a major player in the Indian Ocean trade system [6]. The sheer quantity of these glass beads is consistent with a very influential place. While bronze and other copper-based objects were recovered at Mapela, so far no gold objects have been recovered. Given that Mapela is just less than 20 kilometres away from the Gwanda-West-Nicholson gold belt, it is only a matter of time before gold is found. Another possibility is that some of the gold may have been looted in the late 19^th^ and early 20^th^ centuries when virtually all the stone-walled sites were looted for their gold. Even today, the ghost of past plundering rears its ugly head in the form of multiple illicit trenches in parts of Mapela with evidence of metallurgical slags. Herbert [38] has argued that bronze was more valuable in the African value system when compared with gold. Nevertheless we should not forget that stone walls and class distinction are the most important elements of the Zimbabwe culture which, as we have seen, is evident at Mapela.

**Figure 15:**
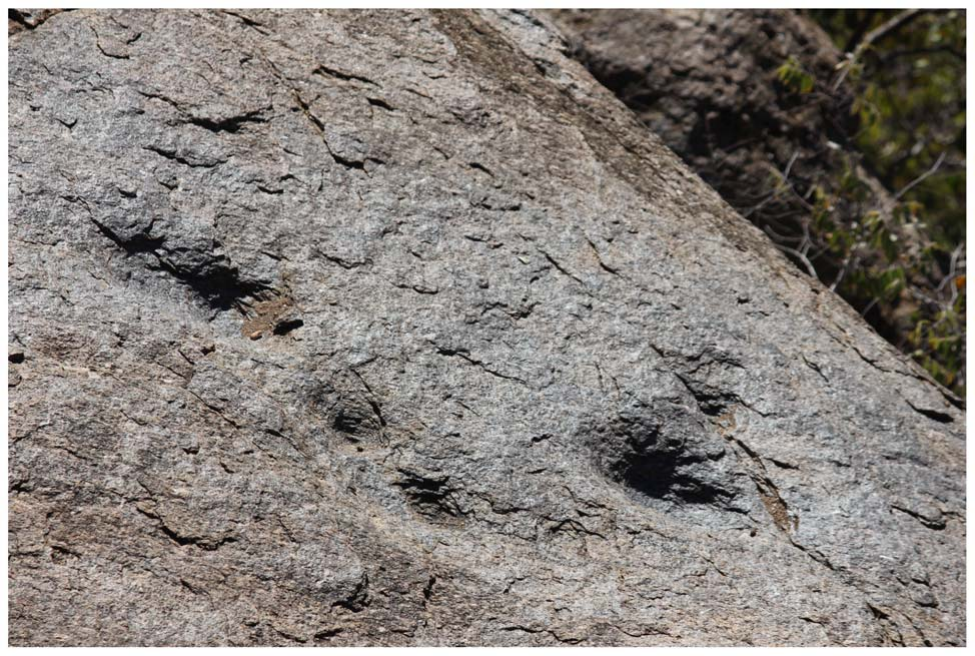
Cupules located on the eastern edge of the north-facing cliff at Mapela.

Recently, the cultural practice of rainmaking has been placed deep within the debate about early state formation in southern Africa. Murimbika [39] and Schoeman [40] speculate about the contribution of rainmaking to the evolution of the state based at Mapungubwe in the early 13^th^ century CE. Specifically, it is argued that before the 13^th^ century CE, rainmaking took place in the natural environment, away from homesteads. However, this practice changed during the Transitional K2 period when certain individuals appropriated rainmaking control on Mapungubwe Hill and used it as springboard to political power. The signature of rainmaking includes steep-sided hills difficult to access, as well as infrastructure such as rock tanks and artificial cupules. Huffman [21] also argues that rainmaking control was associated with burning houses and granaries as part of the ritual process linked with rainmaking. Mapela possesses this signature of rainmaking in abundance. At 90 metres high and with very sheer cliffs, Mapela is steep sided and difficult to access, taking on average at least an hour to get to the summit. Furthermore, it contains rock tanks and cupules at various levels, from the hilltop to the lower terraces (Figures 7 and 15). Finally, there is evidence of heavy burning in the sequence from the K2 period onwards. Given the early crystallisation of the Zimbabwe culture attributes at Mapela when compared with the middle Limpopo valley, it seems that control of rainmaking appeared here much earlier. However, as Chirikure *et al*. [9] have argued, the institution of rainmaking was entrenched in various Shona communities and, like stone walls, participation in long-distance trade and settlement on raised ground, cannot be attributed only to one point on the landscape.

**Figure 16:**
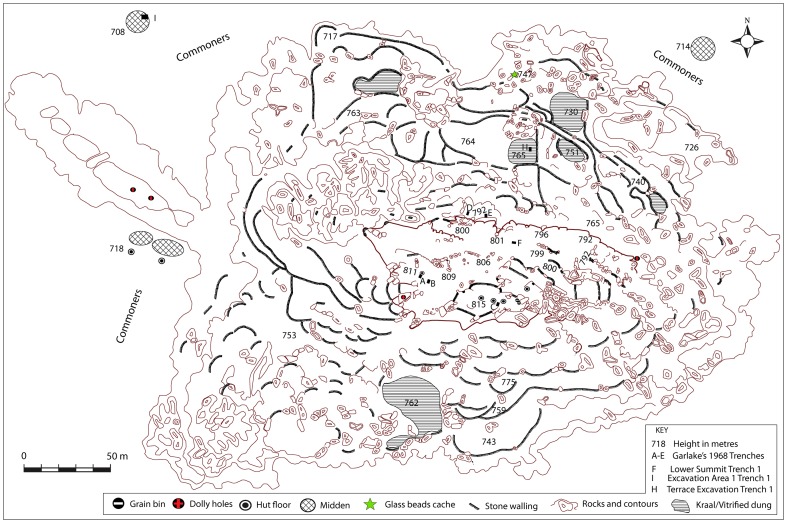
Map of Mapela (CE1055–1400), its size and significant number of stone walls.

**Figure 17:**
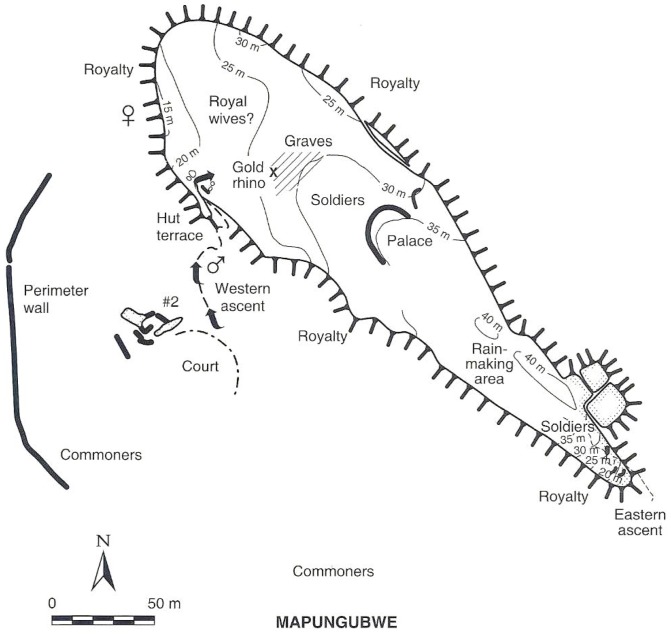
Map of Mapungubwe (CE1220–1290) (after Huffman, 2007): Note the limited number of walls and small size when compared with Mapela.

Having demonstrated the presence of élite Zimbabwe culture attributes from the K2 period onwards, emphasis now shifts to the Mapungubwe occupation. Just as Mapela was much larger than K2, it was also bigger than Mapungubwe. Figures 16 and 17 place side by side, respectively, the complete maps of Mapela and Mapungubwe, and decisively show that the former is considerably bigger than the latter. This incontrovertibly refutes Garlake's initial assumption that Mapela is much smaller than Mapungubwe. While the Mapungubwe hilltop is elongated with only one terrace platform (Figure 17), that of Mapela is much wider with substantially more walling. Fundamentally, where Mapela Hill is heavily terraced along most of its contours, Mapungubwe is not. The enormous size of the walls at Mapela (Figure 4), and the labour evidently invested in constructing terraces, far exceeds that reported for the Leopard's Kopje sites in southern Africa. On the basis of this new data, it is undeniable that southern African archaeologists relied too much on Garlake's account without visiting the site themselves. The result was that Mapela was made to suit different explanatory frameworks while using erroneous assumptions.

**Figure 18:**
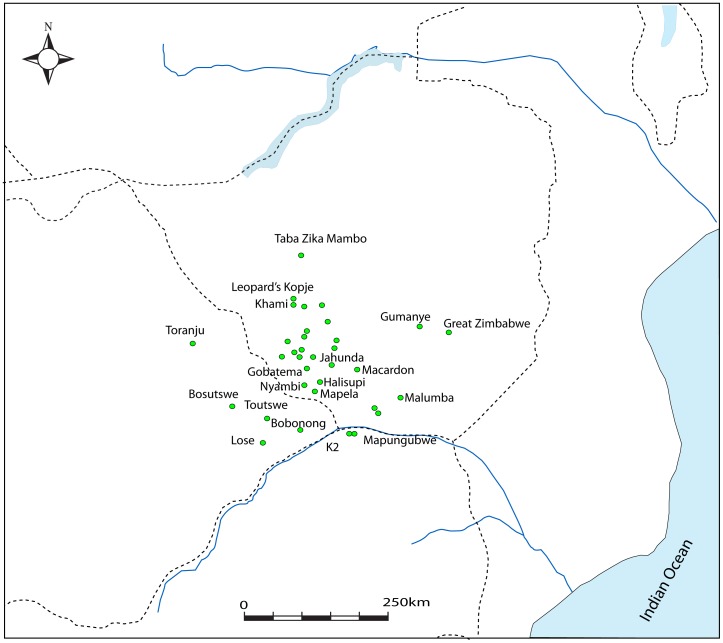
Mapela and some chronologically overlapping sites with Leopard's Kopje Phases I and II pottery.

Figures 16 and 17 credibly and clearly demonstrate that Mapela is larger than both K2 and Mapungubwe. Therefore, an earlier manifestation of the Zimbabwe culture at Mapela fundamentally requires a rethink of the beginnings of socio-political complexity in southern Africa. If the dominant framework was not already problematic because of its blinkered focus on a few sites on the landscape, we would have argued that Mapela and perhaps rightly so, is the largest known and first Zimbabwe culture capital in southern Africa. The amount of stone walling on Mapela, abundant glass beads and evidence of class distinction makes it unlikely that it was under K2 or Mapungubwe. However, as Chirikure *et al*. [9], [25] have incontrovertibly demonstrated, the individual elements of dry stone wall construction, élite occupation of hilltops, class distinction, participation in long-distance trade, rainmaking and the construction of solid *dhaka* floors singly and in combination are widespread in south-western Zimbabwe and adjacent regions. The distances between Taba Zika Mambo in the Zimbabwean Midlands, Mapungubwe in the Shashe-Limpopo and Jahunda in south-western Zimbabwe, where all these features are expressed, makes it difficult to understand how given the logistical limitations of the time, any one of these entities could have dominated the whole landscape politically and economically (Figure 18).

To move away from the linear view which is, in any case, troubled with misattribution, a framework provided by Actor–network theory (ANT), was exploited to account for the rise of socio-political complexity in southern Africa. ANT simultaneously considers the human aspects as well as ideology and relationships between multiple entities on the landscape. In ANT, actors are combinations of symbolically invested things, identities, relations, inscriptions, and networks capable of nesting within other diverse networks [41]; [42]. Networks are processual, built activities, performed by the actants out of which they are composed. Because of interlinkages, networks are local, variable, and contingent since there is no disjunction between agency and structure. An actor network is the act linked with all of its influencing factors in building a network [41]; [43].

Because of the existence of many widely separated but chronologically overlapping Leopard's Kopje communities in south-western Zimbabwe, south-central Zimbabwe, north-eastern Botswana and northern South Africa (middle Limpopo valley) (Figure 18) it is prudent to consider them as various actors that are coeval on the landscape. Although these actors were independent, they interacted and shared the same ideology, while participating in local, regional and international trade. Broadly speaking, they formed a network between themselves and within themselves. For example, the presence of Eiland ceramics at Little Mapela, at Mapungubwe, and in the Tswapong Hills demonstrates interaction between various communities from Tzaneen near the Kruger National Park to the edge of the Kalahari. Wilmsen *et al*. [44] demonstrated through ceramic petrography that late first millennium and early second millennium AD communities in northern and northwestern Botswana were interacting with those in the Shashe-Limpopo area some 600 kilometres away. The links may have been direct or through intermediaries, but it is always likely that they were based on trade and exchange of various goods and commodities. Historically, various Tsonga communities obtained trade goods from Delagoa Bay and travelled considerable distances inland to obtain iron hoes, tin and other local resources in exchange for glass beads and other commodities [45].

Furthermore, the similarity in glass beads over time and the almost identical frequency suggests that all the actors participated in the network with no single entity monopolising the trade. To use Renfrew and Cherry's [46] interpretation, these actors were also competing and possibly conflicting with peers, some of whom were more successful than others, creating a network of interlinked entities during the Early (Leopard's Kopje) and Late Zimbabwe culture. In some cases, the bigger settlements were networked with smaller villages and towns within a short distance. For instance, Mapela is surrounded by extensive smelting villages near Nyambi Hill, and smaller settlements such as (Little) Halisupi located about 20 kilometres north-east of Mapela. Halisupi comprises a walled hilltop site and a flat area with middens containing Leopard's Kopje pottery, slag, shell beads, animal bone and vitrified dung. Similarly, Mapungubwe was surrounded by smaller settlements [21] just as Bosutswe [47], Great Zimbabwe [48], and other major centres. Given that all these were Shona communities with shared cultural values, they were strongly networked. It is therefore unlikely that they were unaware of one another. As such, if innovations started in one place, they would have been easily copied, making it difficult to identify the first palace, first rainmaking hill, first gold object and first stone wall, given the limits of absolute dating techniques.

Unlike the traditional model, which ignores all these networks and unnecessarily robs the region's past of dynamism, ANT places these entities at the centre of societal change. The result is that southern Africa around CE1000 had multiple socio-politically complex entities that competed, conflicted and interacted with one another [49]. In view of these chronologically overlapping but different communities with uniform cultural features, it does not make sense to argue that the origin of the Zimbabwe culture is at K2 and Mapungubwe. Rather, it is perhaps far closer to the truth to argue that the roots of the Zimbabwe culture were sowed during the Leopard's Kopje Phase I period, and that during the same time, various Shona people resident in southern Zambezia participated in long-distance trade, in local trade , and developed a number of cultural attributes visible on the whole landscape. With time, these communities started to build very elaborate dry stone-walled palaces epitomised early on by Malumba, Mapela, Halisupi and Mapungubwe, and later by the grand capitals such as Tsindi, Great Zimbabwe and Khami. Even during this early period, many actors were at play on this landscape, which explains the hundreds of Leopard's Kopje-, Great Zimbabwe- and Khami-type settlements dispersed between the Indian Ocean and the Kalahari. Monroe [50] discusses the agency of indigenous political entrepreneurs who spearheaded early state formation across the African continent. Based on the evidence presented here, it seems that the various Leopard's Kopje actors were entrepreneurs who competed with one another, resulting in the establishment of various political centres that were coeval.

This interpretation is adequately supported by Shona historical and anthropological data which demonstrate the existence of robust networks at various levels, from the small- to the large-scale [51]; [52]. According to Beach [53], in terms of political networks, various Shona groups had chiefdoms most of which were related through common ancestry. Some states such as the Mutapa were comprised of various smaller chiefdoms led by houses that alternated the Mutapa kingship following rotational succession. When they were not capitals, their status was transient, such that they could become provincial or district centres, and even capitals again. This practice created a series of capitals associated with various Mutapa and Buhera leaders respectively in northern and central Zimbabwe [52]. Within the Mutapa state, there are a number of ruins, such as Mutota, Kasekete, Nowedza, Matope and many others, which are associated with former Mutapa kings. However, because power shifts within the network were short-term events, it is often difficult to date them using radiocarbon [54]. Mudenge [37] argues that the Mutapa kings did not monopolise external trade since they allowed different actors to participate. However, they levied taxes on commodities passing through their land. As a result, the theory of rotational succession dictates, as conditioned by context, that the various actors or peers on the landscape would through historical circumstances change their position, resulting in a very dynamic history. Not surprisingly, Beach [52] believes that Mapela was a capital of an independent polity, just as in early Khami, Great Zimbabwe, Mapungubwe, Jahunda, and many others. Some communities or actors became more successful than others, while new ones rose in place of the old. For this reason, it makes sense to associate the evolution of the Zimbabwe culture initially with the multiple Leopard's Kopje Phase I sites, some of which developed into the Leopard's Kopje Phase II, and eventually into the Zimbabwe culture.

It is prudent to pose the question of why these innovations in socio-political organisation are associated with the Leopard's Kopje period after CE900 onwards. The overarching influence of demography is one critical variable that has been overlooked in studies of the Iron Age of southern Africa. Shennan [55] has shown that social and technological innovations tend to be successful in contexts with large populations. To consider the impact of population change at a qualitative level, research was carried out using the database of Iron Age sites held by the Museum of Human Sciences in Harare. The entries in the database indicate that in south-western Zimbabwe, there are more sites dating between CE900 and 1300 than in the preceding period. A focused area study of settlement patterning and succession in the Maramani area of south-western Zimbabwe by Manyanga [28] identified more Leopards' Kopje sites when compared to Early Iron Age sites. Indeed, the distribution maps published by Huffman [24] show that there are more Mapungubwe sites when compared with K2 and Zhizo sites in the middle Limpopo valley. Because site density is a useful proxy for population size, it is undeniable that the late first- and early second millennium CE interface is associated with increasing populations and, by extension, increasing innovations. Sceptics may argue that often Early Iron Age sites are often not easily visible, but across southern Africa the evidence points to increased village sizes from CE700 onwards [22] which climaxes in the very large villages of the late first millennium CE, such as Swart Village, Nyambi and Schroda. From then on, the sites became increasingly larger, with populations numbering in the thousands in the case of places such as Mapela, Mapungubwe, Great Zimbabwe, and many others. Just as Shennan [55] has eloquently argued in a different context, the advent of the Zimbabwe culture in the early Leopard's Kopje period is a consequence of demographic growth and increased range of contact evident from the distribution of many Leopard's Kopje sites, most of which still remain unstudied in southern Africa. In this big pool it would be somewhat inane, even preposterous, to argue that it is possible to identify the first palace in the Zimbabwe culture, the first *dhaka* floors, and the first rainmaking hill. Rather, it is possible to see a network of actors who exhibit shared cultural traits occasioned by various forms of interaction.

From a comparative perspective, it is important to briefly consider the emergence of socio-political complexity in the world. In the Middle East around 4000 BCE various city states of Sumer were actors or peers who networked and often competed with one another [56]. In this part of the world, socio-political complexity did not start with only one polity, such as Ur. Instead, the evidence shows the presence of multiple communities at places like Uruk, with more or less identical attributes to those of Ur and others. Similarly, the various Aegean polities were interacting with one another through trade and exchange, conflict and other mechanisms, resulting in the rise of complexity in the area [46]. In the New World, various Mayan polities also interacted with one another forming a network over time that resulted in the evolution of early states [57]. As Yofee [56] argues, most archaic states were much smaller in territorial extent than those of today, a view which is consistent with the many Leopard's Kopje polities (if one may call them that) in our region from CE900 onwards. The emphasis in these parts of the world is not on finding the earliest city state of Sumer, neither is it on finding the earliest Mayan city state, for to do so would be pointlessly investing energy in finding the first grain of sand to be deposited in an ocean. Rather, the focus is on combining evidence to understand how various polities or actors may have interacted, resulting in continuity and change. If such an approach is adopted in southern Africa, it may be possible to learn more about the dynamism of the Leopard's Kopje and its apogee, the Zimbabwe culture.

## Conclusion

On the basis of the evidence presented in this paper, it is clear that Mapela occupies an important space in debates on early state formation in southern Africa. Fundamentally, the ceramics, glass beads, chronometric and architectural evidence suggest that Mapela had fully developed attributes of the Zimbabwe culture during K2 times. Furthermore, the ideology of class distinction and sacred leadership at Mapela antedates that at K2 and Mapungubwe. On this evidence neither K2 nor Mapungubwe qualify to be the region's incipient Zimbabwe culture centres. Of course Mapungubwe has yielded an impressive array of gold objects which are yet to be found at Mapela. However, on the basis of cultural precedence, Mapela had a fully developed Zimbabwe culture much earlier than Mapungubwe. The thousands of glass beads found at Mapela show that it was a principal player in long-distance trade. Mapela has also been shown to be considerably larger than Mapungubwe. Therefore, it would be unreasonable to conclude that a later site with far fewer dry stone walls is more important than one with more, if both the sites are within the formation of the Zimbabwe culture period.

A combination of historical evidence with Actor−network theory and its variants such as peer polity interaction, suggests that towards the early second millennium CE, various Shona communities resident in the region bound by the Indian Ocean to the east and the Kalahari to the north underwent a series of transformations ideologically, culturally, economically and politically. The seeds of these transformations were sowed during the Leopard's Kopje Phase I and were elaborated during the Zimbabwe culture period. The presence of various actors exposes markedly the undesirability of models which fix the birth of early state formation at single points in time. It is far more prudent to argue that various communities exhibiting identical cultural practices all contributed and participated in early state formation. As such, early state formation did not start at Mapungubwe; neither did it start at Great Zimbabwe or Khami. Instead, it started with the many Leopard's Kopje sites which show evidence of the expression of Zimbabwe culture in south-western Zimbabwe and adjacent regions. The thinking that the evolution of the Zimbabwe culture started at Mapungubwe through Great Zimbabwe to Khami must now surely be revised. This is because as elsewhere, it is impossible to pinpoint the start of an innovation which is widely expressed across a vast stretch of land by using only a few sites. Certainly the Zimbabwe culture started with the Leopard's Kopje, which evolved into the Zimbabwe, but the mechanisms of such continuity and change are unknown. Huffman ([21]: 49) has honourably pointed out that the status of various Leopard's Kopje walls and sites in south-western Zimbabwe and north-eastern Botswana has not been fully considered. We have presented chronological and material culture evidence from one site in that area, which indicates that the Zimbabwe culture crystallised during K2 times, making it unlikely that Mapungubwe is the first Zimbabwe culture capital, as has been generally assumed until now. The evolution of early state formation in our region followed multiple trajectories mediated by context-specific situations. In the process, some communities failed where others succeeded, with reversals taking place at different intervals. The net sum is that there are hundreds of widely separated and chronologically overlapping actors belonging to the Leopard's Kopje and Zimbabwe cultures. The major lesson for global archaeology is that from time to time it is absolutely critical to update our knowledge of any given area through continuous fieldwork and new questions. Indeed, some archaeological truths started as opinions which were not verified on the ground, but were then repeated widely thereby becoming ‘truths’. These truths must be tempered with new information and insights to create more dynamic pasts.
